# Risk factors for asthma exacerbations

**DOI:** 10.1016/j.jacig.2025.100520

**Published:** 2025-06-21

**Authors:** Lauri Nordman, Iida Vähätalo, Leena E. Tuomisto, Onni Niemelä, Minna Tommola, Lauri Lehtimäki, Pinja Ilmarinen, Hannu Kankaanranta

**Affiliations:** a Faculty of Medicine and Health Technology, Tampere University, Tampere, Finland; b Department of Respiratory Medicine, Seinäjoki Central Hospital, Seinäjoki, Finland; c Department of Laboratory Medicine, Seinäjoki Central Hospital, Seinäjoki, Finland; d Department of Respiratory Medicine, Hospital Nova of Central Finland, Jyväskylä, Finland; e Allergy Centre, Tampere University Hospital, Tampere, Finland; f Department of Internal Medicine and Clinical Nutrition, Krefting Research Centre, Institute of Medicine, University of Gothenburg, Gothenburg, Sweden

**Keywords:** Adult-onset, asthma, exacerbation, risk factor, prevalence, follow-up, GERD, unplanned respiratory-related health care visit, medication, comorbidity

## Abstract

**Background:**

The Global Initiative for Asthma (GINA) advises evaluation of risk factors for exacerbations. Patients with adult-onset asthma often have a problematic disease presentation with comorbidities. Not much information exists about the prevalence of exacerbation risk factors in adult-onset asthma patients.

**Objective:**

We evaluated the prevalence of exacerbation risk factors listed in the 2023 GINA Report and their association with exacerbations in patients with adult-onset asthma.

**Methods:**

In the Seinäjoki Adult Asthma Study, 203 patients with adult-onset asthma were followed for 12 years. Data were available for 17 of the 21 GINA risk factors. Exacerbation was defined as an unplanned health care visit with asthma exacerbation mentioned in the medical health records. ClinicalTrials.gov NCT02733016.

**Results:**

On average, patients had 3.8 asthma exacerbation risk factors, with poor adherence (54.7%), chronic rhinosinusitis (54.2%), and smoking (52.7%) being the most common. Four patients (2.0%) had no risk factors. Oral corticosteroid use and the number of exacerbations increased with the number of risk factors. Exacerbations were positively associated with gastroesophageal reflux disease (odds ratio [OR], 5.0; 95% confidence interval [CI], 1.7-15.1; *P* = .004) and age >50 years (OR, 2.3; 95% CI, 1.1-4.8; *P* = .021) in univariate analyses. After adjustments by sex, pack years, and body mass index, only gastroesophageal reflux disease remained as a statistically significant risk factor (OR, 4.5; 95% CI, 1.5-14.0; *P* = .009).

**Conclusions:**

Exacerbation risk factors are common in patients with adult-onset asthma, and the value of identifying a single risk factor may be low. Patients with multiple risk factors and/or gastroesophageal reflux disease should be emphasized in clinical practice.

Asthma is commonly regarded as a childhood disease, but adult-onset asthma is also common and is associated with high medication use, severe respiratory symptoms, low remission rate, and high mortality rate.^1^ Although successful treatment and exacerbation prevention are crucial factors when addressing asthma patients’ quality of life and health care costs, not much information on the disease control of adult-onset asthma exists.^2^

Exacerbations may be associated with poor adherence to inhaled corticosteroids (ICS) and high use of short-acting β_2_-agonist therapy,^3^, ^4^, ^5^ although contradicting evidence has been presented.^6^ While the prevention of exacerbations often focuses on appropriate medical therapy, other factors such as comorbidities, patient demographics (eg, education), and control of triggering factors should also be taken into consideration.^7^ Previous studies have shown predisposing factors for exacerbations to include female sex, obesity, smoking, air pollution exposure, low adherence to therapy, incorrect medication use, and comorbidities such as gastroesophageal reflux disease (GERD) and chronic rhinosinusitis.^3^^,^^8^, ^9^, ^10^, ^11^, ^12^ In addition, viral respiratory infections have been reported to be a common trigger for exacerbations, and effective detection and prevention of these infections could greatly reduce the number of exacerbations.^3^^,^^13^^,^^14^

The Global Initiative for Asthma Report 2023 (hereafter the GINA Report) presents numerous risk factors for exacerbations without addressing their comparable importance.^15^ A detailed evaluation of the prevalence and importance of different risk factors is lacking. Mortality and costs related to chronic asthma are largely associated with exacerbations, and new preventive approaches are needed.^14^ This study was conducted to assess the prevalence and relative importance of asthma exacerbation risk factors, as reported in the GINA Report, in patients with adult-onset asthma, and to evaluate which risk factors we should concentrate on when optimizing exacerbation prevention.

## Methods

### Study design and patients

The current study is a part of the Seinäjoki Adult Asthma Study, a prospective single-center (Seinäjoki Central Hospital, Seinäjoki, Finland) 12-year follow-up study containing 256 adult patients (≥15 years) with a diagnosis of new-onset adult asthma. All patients with a new-onset asthma diagnosis by a respiratory specialist, a confirmation of diagnosis by lung function measurements showing reversible obstruction, and symptoms of asthma were included between 1999 and 2002. Patients with comorbidities, smoking history, or other lung diseases were not excluded. The diagnostic criteria, inclusion/exclusion criteria, and study protocol have been published elsewhere (see Table E1 in this article’s Online Repository available at www.jaci-global.org).^16^ Participants provided written informed consent to the study protocol approved by the ethics committee of Tampere University Hospital, Tampere, Finland.

The study population consisted of 203 patients (79.3%) who attended their 12-year follow-up. The remaining 53 patients (20.7%) from the original cohort were excluded. Data concerning things like medication, comorbidities, and symptoms were collected both at baseline and at 12-year follow-up visits. Further details on data collection are provided in the Methods section and in Fig E1 available in the Online Repository at www.jaci-global.org. Medication information and asthma-related visits (primary, secondary, and private health care) were collected from the medical records for the entire 12-year follow-up period.^17^

### Exacerbation risk factor assessment

In the 2023 GINA Report, box 2-2 addresses asthma exacerbations and their risk factors.^15^ Seven risk factor categories consisting of 21 individual risk factors are listed (see Table E2 in the Online Repository available at www.jaci-global.org). The categories and the individual factors are separately addressed as independent risk factors in the exacerbation risk assessment of this study. Of the 21 individual risk factors, 17 were examined. Unexamined individual risk factors were ICS not being prescribed (all patients were prescribed ICS), incorrect inhaler technique (not systematically reviewed), e-cigarette use (study period before e-cigarette era), and air pollution exposure (insufficient data, low-pollution area^18^).

All patients were evaluated for categorical (0-7) and individual risk factor (0-17) scores. The criteria for scoring points (ie, risk factor definitions) are provided in Table E3 in the Online Repository available at www.jaci-global.org and are based on the information and risk factor definitions provided by the GINA Report. Forced expiratory volume in 1 second (FEV_1_) was addressed at baseline. Obesity, GERD, blood eosinophils, fractional exhaled nitric oxide (Feno), IgE antibodies, smoking, psychological and socioeconomic problems, and food allergies were addressed at follow-up. Short-acting β_2_-agonist and ICS use, ICS adherence, bronchodilator responsiveness, chronic rhinosinusitis, pregnancies, prior exacerbations, and intensive care unit visits were addressed throughout the 12-year follow-up period. Missing values (<1.6%) were interpreted as the criterion’s not being fulfilled, therefore resulting in no points. The GINA Report’s risk factor categories include various numbers of individual risk factors (Table E2). Categorical points required ≥1 individual factors positive in the category. When addressing earlier severe exacerbations as a risk factor, a severe asthma exacerbation was defined by the GINA Report as ≥1 unscheduled hospital admissions for asthma or ≥1 course of oral corticosteroid (OCS) therapy for asthma. Hospital admissions were gathered from medical records, and OCS use was self-reported. As risk factors were evaluated over a long period, “severe exacerbations in the last 12 months” could not be determined. Therefore, all exacerbations during the follow-up resulted in a risk factor point. Smoking a pack of cigarettes a day for 1 year constituted 1 pack year.

### Risk factor scores and adverse outcomes

Binominal regression analyses were conducted to study the associations between risk factors and exacerbations (0 vs ≥1). The association was examined separately for categorical and individual risk factors. Asthma exacerbation as an outcome was defined as an unplanned health care visit with asthma exacerbation mentioned in the medical health records. Exacerbation history and the individual risk factors in that category were left out of the regression analyses because only exacerbations observed during the follow-up period were available for analysis, and exacerbations that occurred before baseline or after follow-up visit were not available in this study.

Additional exploratory analyses were conducted to examine the connection between exacerbation risk factors and other causes resulting in worsening asthma symptoms. Upper respiratory tract infections (URTIs) were considered to be a common misdiagnosis for exacerbations and therefore were included in the analyses. An unplanned respiratory-related health care visit (URHV) was defined as an unplanned health care visit regarding URTIs and/or asthma exacerbations based on medical records.^17^ The association between exacerbation risk factors listed in the GINA Report and URHVs (0 vs ≥1) was evaluated.

Receipt of OCS therapy during the 12-year follow-up period was evaluated by categorical and individual risk factor scores. Annual and total OCS use were obtained from the Finnish Social Insurance Institution, which records all reimbursed medication purchases from any Finnish pharmacy.

### Statistical analyses

The results are shown as mean (standard deviation [SD]) or median (interquartile range). Categorical variables were described as frequency rates and percentages. To compare study population characteristics at baseline and follow-up, statistical significance was evaluated by paired samples *t* test for normally distributed continuous variables, related samples Wilcoxon for nonnormally distributed continuous variables, and McNemar test for categorical variables. Normality in continuous variables was evaluated by visual inspection of distribution.

Univariate and multivariable binary logistic regression analyses were performed to assess factors associated with asthma exacerbations. The categorical covariates we used included sex, age, body mass index (BMI), pack years, and the 6 categorical and 15 individual risk factors examined in this study. All regression analyses regarding asthma exacerbation risk factors were conducted with the patients without the risk factor in question as the reference group.

*P* ≤ .05 was regarded as statistically significant; associations are presented as odds ratios (ORs) and 95% confidence intervals (CIs). Statistical analyses were performed by SPSS Statistics v28.0.1.0 software (IBM, Armonk, NY).

## Results

### Patient characteristics

Most of the study population comprised women (58.1%). On average, patients gained weight during the 12-year follow-up. Lung function improved while blood eosinophil counts and total IgE levels decreased. In total, 68 patients (33.5%) experienced ≥1 exacerbations, and 158 patients (77.8%) had ≥1 URHVs during follow-up. The characteristics of the study patients are listed in Table I.

**Table I:** Patient characteristics at baseline (at diagnosis) and at 12-year follow-up (n = 203)

Characteristic	Baseline	Follow-up	*P* value
Age (years)	46 (14)	58 (14)	<.001
Male sex, no. (%)	85 (41.9)	85 (41.9)	
BMI (kg/m^2^)	27.1 (24.2-29.8)	28.1 (24.4-31.3)	<.001
Smokers (including ex-smokers), no. (%)∗	103 (50.7)	107 (52.7)	.125
Smoking history (pack years)†	11 (5-20)	16 (7-30)	<.001
Pre-BD FEV_1_ (%)	83 (71-92)	86 (76-96)	<.001
Pre-BD FVC (%)	90 (80-100)	96 (87-106)	<.001
Pre-BD FEV_1_/FVC	0.75 (0.69-0.80)	0.73 (0.66-0.79)	<.001
Post-BD FEV_1_ (%)	88 (77-99)	90 (80-98)	.013
Post-BD FVC (%)	94 (82-102)	98 (88-107)	<.001
Post-BD FEV_1_/FVC	0.79 (0.75-0.85)	0.75 (0.69-0.81)	<.001
Blood eosinophils (10^9^/L)	0.28 (0.15-0.42)‡	0.16 (0.10-0.27)	<.001
Total IgE (kU/L)	84 (35-174)	61 (24-163)	.046
Daily ICS use, no. (%)	16 (8.0)	155 (76.4)	<.001
DL_CO_ (%)	97 (19)	92 (19)	<.001
DL_CO_/VA (%)	100 (17)	94 (17)	<.001
AQ20 score	7 (4-10)	4 (2-7)	<.001

Data are presented as nos. (%), means (SDs), or medians (interquartile ranges). Statistical significance was evaluated by paired-sample *t* test or by related-sample Wilcoxon or McNemar test. *AQ20,* Airways questionnaire 20; *BD,* bronchodilator; *DL*_*CO*_*,* diffusing capacity; *DL*_*CO*_*/VA,* DL_CO_ adjusted by alveolar volume; FVC, forced vital capacity.

∗ During 2000-2013, nine patients started smoking (8 of whom restarted), and 14 patients quit smoking, 3 of whom also started during 2000-2013.

† Smoking a pack of cigarettes a day for 1 year constituted 1 pack year.

‡ Total of 15.6% with ongoing steroid treatment.

### Prevalence of asthma exacerbation risk factors

Data of all 203 patients were included in the categorical risk score evaluation, while in the case of individual risk factors, data concerning ICS use (n = 181), adherence (n = 181) and Feno (n = 193) were not available for all. The majority received a point from the following risk factor categories: other medical condition (79.3%), medication (57.1%), and exposure (55.7%). The number of patients scoring a point in each risk factor category is shown in Table II. The most common individual risk factors were poor adherence (54.7%), chronic rhinosinusitis (54.2%), and smoking (52.7%).

**Table II:** Prevalence of reviewed categorical and individual asthma exacerbation risk factors in patients with adult-onset asthma

Characteristic	No. of patients	Prevalence, no. (%)
**Categorical risk factors**		
Other medical conditions	203	161 (79.3)
Medications	203	116 (57.1)
Exposures	203	113 (55.7)
Exacerbation history	203	76 (37.4)
Lung function	203	52 (25.6)
Psychosocial	203	50 (24.6)
Type 2 inflammatory markers	203	40 (19.7)
**Individual risk factors**		
Poor adherence	181	99 (54.7)
Chronic rhinosinusitis	203	110 (54.2)
Smoking	203	107 (52.7)
≥1 severe exacerbation in last 12 months∗	203	75 (36.9)
Obesity	203	71 (35.0)
Inadequate ICS	181	54 (29.8)
Major psychological or socioeconomical problems	203	50 (24.6)
Confirmed food allergy†	203	41 (20.2)
High BD responsiveness	203	39 (19.2)
Higher blood eosinophils	203	35 (17.2)
High SABA use	203	21 (10.3)
Low FEV_1_	203	21 (10.3)
Pregnancy	203	18 (8.9)
GERD	203	16 (7.9)
Elevated Feno	193	10 (5.2)
Allergen exposure if sensitized	203	10 (4.9)
Ever intubated or in intensive care unit for asthma	203	1 (0.5)

Factors presented by GINA Report.^15^ Having ≥1 individual risk factors in the risk factor category resulted in a categorical risk factor point. Definitions for categorical and individual risk factors based on GINA Report are presented in Table E3. *BD,* Bronchodilator; *SABA,* short-acting β2-agonist.

∗ All exacerbations during 12-year follow-up period were considered.

† Defined as sensitization to at least one food allergen component because there are no data to be considered regarding allergy symptoms or food allergy diagnosis.

**Fig 1:**
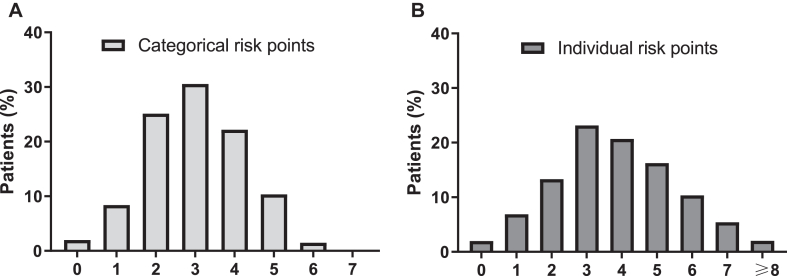
Categorical and individual risk factor scores. Distribution of categorical **(A)** and individual **(B)** risk factor scores among adult-onset asthma patients during 12-year follow-up. Categorical (0-7) and individual (0-17) risk factors are based on GINA Report.^15^

Four patients (2.0%) had zero risk factors, while most patients (78%) had ≥3 individual risk factors. The average categorical and individual risk factor scores were 3.0 and 3.8, respectively. The categorical and individual risk scores are shown in Fig 1 and in Fig E2, which is available in the Online Repository available at www.jaci-global.org.

### Risk factor scores, exacerbation frequency, and OCS use

**Fig 2:**
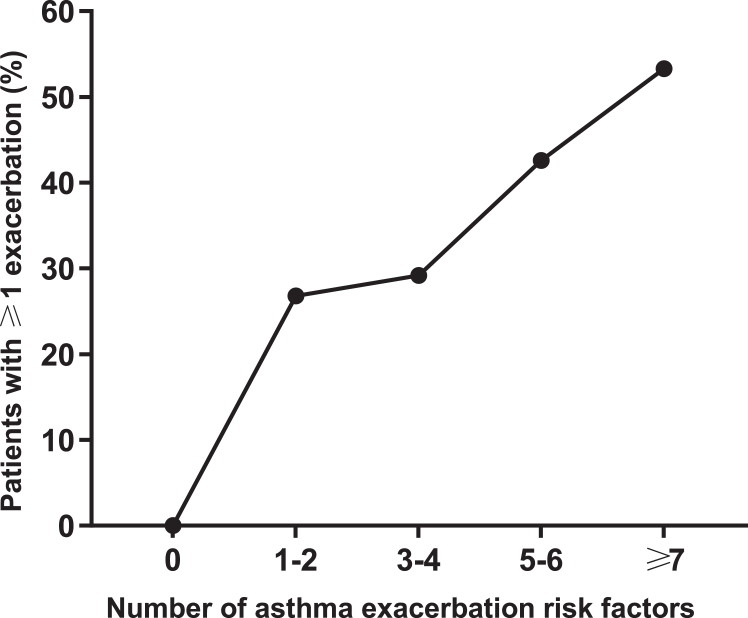
Prevalence of experiencing ≥1 asthma exacerbations by individual risk factor scores during 12-year follow-up. Asthma exacerbation was defined as unplanned health care visit with asthma exacerbation mentioned in medical health records.

On average, the proportion of patients experiencing ≥1 asthma exacerbations, exacerbation frequency, and OCS use increased with higher categorical (see Table E4 in the Online Repository available at www.jaci-global.org) and individual risk factor scores (Fig 2, Table III). Patients with a high individual risk factor score (≥4) had a higher incidence of experiencing an exacerbation during follow-up compared to patients whose individual risk factor score was below average (3.8): respectively, 37.8% and 28.3%. OCS use and exacerbation frequency by individual risk factor scores are presented in Table III.

**Table III:** Exacerbation frequency and OCS use by individual risk factor scores

Characteristic	Asthma exacerbation risk factor score								
	0	1	2	3	4	5	6	7	≥8
No. of patients	4 (2.0)	14 (6.9)	27 (13.3)	47 (23.2)	42 (20.7)	33 (16.3)	21 (10.3)	11 (5.4)	4 (2.0)
Patients with ≥1 exacerbations during follow-up	0	4 (28.6)	7 (25.9)	15 (31.9)	11 (26.2)	17 (51.5)	6 (28.6)	5 (45.5)	3 (75.0)
Exacerbations during follow-up	0	0 (0-1.25)	0 (0-1.0)	0 (0-1.0)	0 (0-1.0)	1 (0-4.0)	0 (0-1.5)	0 (0-2.0)	2 (0.25-9.75)
OCS, mg/1 year	0 (0-30)	22 (0-82)	12 (0-131)	64 (0-150)	29 (0-146)	133 (5-423)	109 (7-248)	144 (0-323)	208 (52-666)
OCS, mg/12 years	0 (0-375)	300 (0-998)	150 (0-1500)	820 (0-1800)	375 (0-1813)	1700 (55-4975)	1200 (75-3000)	1800 (0-3820)	2400 (563-7950)

Data are presented as nos. (%) and medians (interquartile ranges). Asthma exacerbation was defined as unplanned health care visit with asthma exacerbation mentioned in medical health records. Dispensed OCS doses were obtained from the Finnish Social Insurance Institution, which records all purchased medication from any Finnish pharmacy.

### Risk factors associated with asthma exacerbations

Univariate binary logistic regression analyses were conducted for all individual (n = 17) and categorical (n = 7) risk factors, pack years, BMI, age, and sex (Table IV, and see Table E5 in the Online Repository available at www.jaci-global.org). GERD (OR, 5.02; 95% CI, 1.67-15.10; *P* = .004) and age (>50 years) (OR, 2.33; 95% CI, 1.14-4.79; *P* = .021) were positively associated with asthma exacerbations. In contrast, poor adherence (OR, 0.5; 95% CI, 0.3-1.0; *P* = .044) was negatively associated with exacerbations. Age (>50 years) and poor adherence lost significance in multivariable binary logistic regression analyses (enter, forward, backward) including sex, age, and 15 individual risk factors as covariates. After adjusting for sex, age, smoking, and obesity using a multivariable logistic regression analysis, GERD (OR, 4.8; 95% CI, 1.6-15.0; *P* = .006) remained a statistically significant risk factor (see Table E6 in the Online Repository). Additional adjustment was conducted replacing smoking and obesity with pack years and categorical BMI, but without a change in the results; GERD (OR, 4.5; 95% CI, 1.5-14.0; *P* = .009) remained a statistically significant risk for asthma exacerbations (Table V). Patients with GERD had higher adherence to ICS therapy than patients without GERD (independent samples *t* test; *P* = .003), with mean adherence 96.3% (SD, 39.6%) and 66.5% (SD, 37.2%), respectively.

**Table IV:** Binominal univariate logistic regression analyses for individual risk factors

Characteristic	≥1 Exacerbations		Exploratory analysis: ≥1 URHV	
	OR (95% CI)	*P* value	OR (95% CI)	*P* value
Female	0.953 (0.528-1.720)	.874	1.625 (0.843-3.132)	.147
>50 years old	2.333 (1.137-4.788)	.021	1.272 (0.627-2.583)	.505
High SABA use	1.564 (0.624-3.917)	.340	1.913 (0.538-6.802)	.316
Inadequate ICS	0.633 (0.316-1.269)	.197	0.582 (0.281-1.208)	.146
Poor adherence	0.530 (0.285-0.983)	.044	0.929 (0.461-1.873)	.838
Obesity	1.125 (0.612-2.066)	.704	1.055 (0.531-2.097)	.878
Chronic rhinosinusitis	1.452 (0.804-2.624)	.216	2.605 (1.326-5.119)	.005
GERD	5.018 (1.667-15.104)	.004	4.894 (0.629-38.067)	.129
Confirmed food allergy	0.903 (0.434-1.881)	.786	0.779 (0.356-1.706)	.533
Pregnancy	0.745 (0.254-2.183)	.591	1.560 (0.432-5.640)	.497
Smoking	1.212 (0.675-2.176)	.521	1.521 (0.789-2.931)	.210
Allergen exposure if sensitized	1.344 (0.366-4.931)	.656	1.216 (0.249-5.934)	.809
Major psychological or socioeconomic problems	0.914 (0.462-1.808)	.796	1.505 (0.670-3.382)	.322
Low FEV_1_	1.251 (0.492-3.182)	.638	1.315 (0.420-4.118)	.638
High BD responsiveness	1.141 (0.549-2.369)	.724	0.610 (0.281-1.326)	.212
Higher blood eosinophils	1.214 (0.569-2.590)	.616	2.000 (0.729-5.486)	.178
Elevated Feno	1.301 (0.354-4.782)	.692	2.681 (0.330-21.773)	.356
BMI∗†				
<25 kg/m^2^	1.000 (reference)		1.000 (reference)	
25-29.99 kg/m^2^	1.186 (0.569-2.475)	.649	0.607 (0.264-1.397)	.241
≥30 kg/m^2^	1.238 (0.592-2.589)	.571	0.788 (0.333-1.861)	.587
Pack years (≥10)∗‡	1.553 (0.841-2.869)	.159	1.717 (0.807-3.655)	.161

Asthma exacerbations were defined as unplanned health care visit with asthma exacerbation mentioned in medical records by clinician. Unplanned respiratory visits were defined as unplanned health care visits regarding URTIs and/or asthma exacerbations based on medical records. All univariate regression analyses regarding asthma exacerbation risk factors were conducted with patients without risk factor in question as reference group. *BD,* bronchodilator; *SABA,* short-acting β_2_-agonist.

∗ Additional exploratory analysis.

† Analysis was conducted with BMI < 25 kg/m^2^ as reference group.

‡ Smoking a pack of cigarettes a day for 1 year constituted 1 pack year.

**Table V:** Binominal multivariable logistic regression analysis of factors associated with asthma exacerbations

Characteristic	OR (95% CI)	*P* value
Female	1.084 (0.556-2.113)	.813
>50 years old	1.944 (0.907-4.168)	.087
GERD∗	4.543 (1.470-14.035)	.009
BMI†		
<25 kg/m^2^	1.000 (reference)	
25-29.99 kg/m^2^	0.988 (0.453-2.152)	.975
≥30 kg/m^2^	1.013 (0.465-2.207)	.974
Pack years (≥10 years)‡	1.436 (0.712-2.896)	.312

∗ Patients without GERD served as reference group.

† Analysis was conducted with BMI < 25 kg/m^2^ as reference group.

‡ Smoking a pack of cigarettes a day for 1 year constituted 1 pack year.

### Exploratory analysis: Risk factor scores and URHVs

On average, the proportion of patients with ≥1 URHVs and the total number of visits during follow-up increased with higher categorical (see Table E7 in the Online Repository available at www.jaci-global.org) and individual risk factor scores (see Table E8 in the Online Repository). Patients with a high individual risk factor score (≥4) had more URHVs during follow-up compared to patients whose individual risk factor score was below average (3.8)—on average, 7.1 and 5.4, respectively. URHVs by individual risk factor scores are shown in Table E8.

Binominal univariate logistic regression analyses were conducted for factors associated with URHVs. Only chronic rhinosinusitis was significantly associated with URHVs (OR, 2.60; 95% CI, 1.33-5.12; *P* = .005) (Table IV). Multivariable regression analyses (enter, forward, backward) performed using sex, age and 15 individual risk factors as covariates did not alter the results. Chronic rhinosinusitis remained statistically significant after adjustment for sex, age, smoking, and obesity (OR, 2.5; 95% CI, 1.27-5.0; *P* = .008) (see Table E9 in the Online Repository available at www.jaci-global.org), and alternatively for sex, age, pack years, and categorical BMI (OR, 2.90; 95% CI, 1.41-5.93; *P* = .004) (Table VI).

**Table VI:** Binominal multivariable logistic regression analyses of factors associated with URHV

Characteristic	OR (95% CI)	*P* value
Female	1.843 (0.876-3.880)	.107
>50 years old	1.222 (0.550-2.713)	.623
Chronic rhinosinusitis∗	2.895 (1.413-5.934)	.004
BMI†		
<25 kg/m^2^	1.000 (reference)	
25-29.99 kg/m^2^	0.567 (0.232-1.388)	.214
≥30 kg/m^2^	0.888 (0.351-2.243)	.801
Pack years (≥10 years)‡	2.132 (0.908-5.006)	.082

∗ Patients without chronic rhinosinusitis served as reference group.

† Analysis was conducted with BMI < 25 kg/m^2^ as reference group.

‡ Smoking a pack of cigarettes a day for 1 year constituted 1 pack year.

## Discussion

We found that asthma exacerbation risk factors are common in adult-onset asthma patients. Patients had an average of 3.8 risk factors for asthma exacerbations, 78% of them had ≥3 risk factors, and only 2% had zero risk factors during the follow-up period. OCS use and exacerbation frequency increased as the risk factor scores cumulated, and GERD was an independent risk factor for future exacerbations.

The GINA Report advises clinicians to assess risk factors for exacerbations (Table E2). The present exacerbation risk assessment included both individual and categorical risk factors, all separately listed by the GINA Report. The aim of this study was to assess the prevalence of asthma exacerbation risk factors in adult-onset asthma patients and their relative importance with regard to exacerbations. In addition, we were set to study the association between the total number of risk factors patients had and the number of exacerbations they experienced during the 12-year follow-up period. Finally, we aimed to study how well the risk factor list provided by the GINA Report can be applied as a tool in exacerbation risk assessment in clinical practice.

Most patients (64.5%) had ≥3 categorical risk factors, indicating that optimal exacerbation prevention requires attention on multiple categories. The most common categorical risk factor was other medical conditions (79.3%), indicating that comorbidities are common in adult-onset asthma. Medications (57.1%) and exposures (55.7%) were also common, highlighting the importance of optimal pharmacotherapy and, in this case, the high smoking prevalence.^14^ Only 4 patients (2%) had zero individual risk factors, while over a third had ≥1 of the following risk factors: poor adherence (54.7%), chronic rhinosinusitis (54.2%), smoking (52.7%), exacerbations during follow-up (36.9%), or obesity (35.9%). All patients were prescribed ICS at the time of asthma diagnosis, but <50% had ≥80% adherence. Unfortunately, poor adherence to prescribed asthma medication is common among asthma patients^19^ even though its connection to adverse and near-fatal outcomes has been reported,^20^ highlighting the importance of adequate patient education. Aligning with our findings, comorbidities such as GERD, chronic rhinosinusitis, and obesity are common features in adult-onset asthma,^21^, ^22^, ^23^ and patients without comorbidities experience fewer exacerbations.^22^ Smoking is still one of the most common and crucial risk factors for exacerbations and hospitalizations.^24^ A significant percentage of adult-onset asthma patients experience exacerbations (33.5% in our cohort), and although earlier studies have assessed different time periods when addressing prior exacerbations, it seems that *all* prior exacerbations carry a high risk of future exacerbations,^25^^,^^26^ emphasizing the importance of primary prevention.

Our findings suggest that patients with childhood asthma and adult-onset asthma should be reviewed differently regarding exacerbations and risk factors. Single risk factors may have a relatively larger effect on children who are usually otherwise healthy and have few comorbidities.^22^^,^^27^ Moreover, the most common reasons behind asthma exacerbations differ when comparing children and adults; for example, children are typically more atopic with higher serum IgE levels and demonstrate a more reversible airway obstruction compared to their adult counterparts.^28^ Adult-onset asthma patients have comorbidities and lifestyle factors (eg, smoking, obesity, work-related exposures) that influence asthma severity and exacerbation frequency.^29^ The prevalence of these comorbidities increases with age^30^ (eg, chronic obstructive pulmonary disease, GERD), decreasing the significance of a single risk factor.

GERD was shown to increase the risk for asthma exacerbations in regression analyses. GERD has been identified as a common feature in patients with severe asthma and frequent exacerbations.^22^^,^^31^ In relation to GERD, a Taiwanese study reported that patients with *Helicobacter pylori* infection exhibited a higher risk of adult-onset asthma than those without *H pylori* infection.^32^ A recent large meta-analysis addressing 32 studies showed an association between GERD and asthma exacerbations that indicated that GERD increases the risk of asthma exacerbations.^10^ A multicenter double-blind randomized study reported a proton pump inhibitor (lansoprazole) to reduce asthma exacerbations, although no improvement in asthma symptoms or pulmonary function was reported.^33^ Although the results of these studies are in line with ours, the findings of studies on GERD in asthma exacerbations remain controversial. However, a bidirectional association between GERD and asthma seems to exist.^34^ We cannot confirm that GERD is causally related with an increased risk of exacerbations, but our results clearly indicate that among evaluated risk factors, even with treatment, GERD (and/or use of proton pump inhibitor medication) is a sign indicating higher risk for asthma exacerbations.

Patients with GERD had high adherence to ICS compared to patients without GERD, although they experienced more asthma exacerbations. Further studies are needed to explore the possibility that high ICS use may cause or worsen symptoms related to GERD. Alternatively, GERD could be the key factor behind worsening asthma symptoms, resulting in higher adherence to asthma medication.

Poor adherence to ICS was associated with reduced risk of exacerbations in univariate regression analysis. This is counterintuitive and in line with our previous findings.^12^ Patients with poor adherence received remarkably lower ICS doses and were often hospitalized less compared to patients with higher adherence. This suggests that patients with poor adherence (<80%) may have had milder asthma compared to patients with higher adherence (≥80%).^12^

Asthma exacerbations can be difficult to distinguish from other causes resulting in similar symptoms, such as URTIs.^35^ Thus, to overcome this, we conducted an exploratory analysis with URHVs (exacerbations and/or URTIs)^36^ used as an outcome. The number of URHVs increased with higher individual risk factor scores. In regression analysis, URHVs were positively associated with chronic rhinosinusitis. These results indicate that the sum score of asthma exacerbation risk factors may also be associated with increased risk of acute visits not due to asthma exacerbation. However, this finding requires further study because it may be confounded by the ability of URTIs to trigger asthma exacerbations.

Although numerous studies addressing asthma exacerbation risk factors have been conducted worldwide, comprehensive reviews of multiple risk factors on adult-onset asthma with long-term follow-up periods have not been previously published. We were able to examine 17 asthma exacerbation risk factors over a 12-year follow-up period. Clinical data were gathered from primary, secondary, and private health care records. Data concerning medication were available on both prescribed and purchased medications. Selection bias was minimized by including all adult patients (≥15 years) with a diagnosis of new-onset adult asthma. Comorbidities, other lung diseases, or any other significant diseases did not result in exclusion, and the study population therefore represents real-life adult-onset asthma patients. Asthma exacerbation as an outcome was defined as an unplanned health care visit with asthma exacerbation mentioned in the medical health records, allowing us to assess the number of experienced exacerbations and to include exploratory analysis in the study. The definition we used also had a clinical aspect: worsened asthma symptoms resulting in an unplanned visit with exacerbation mentioned in the medical health records corresponds to real-life situations and everyday clinical work.

Considering the wide spectrum of risk factors suggested by GINA, some missing data could be considered a study limitation. However, we were able to reliably evaluate 17 (81%) of the 21 risk factors. One weakness of our study is its relatively small study population (n = 203). This may affect the regression analyses, as the prevalence of some single risk factors remains small. However, because most patients had multiple risk factors, the probability of a small study population causing bias regarding risk factor scores is low. After 12 years, 53 patients (20.7%) were lost to follow-up, 22 of whom had died. However, this number of adult patients lost to follow-up during a 12-year period in a clinical study can be seen as low (86.8% response rate among those alive at follow-up). Data comparing baseline characteristics of patients lost to follow-up and not lost to follow-up have previously been published.^2^ A significant difference was found only in prebronchodilator FEV_1_ percentage predicted values.^2^ Some risk factors were not reviewed because of their minor relevance in our cohort, and some risk factor definitions, such as chronic rhinosinusitis and confirmed food allergy, can be seen as incomplete as a result of imperfect data. We did not have annual data on exacerbations; however, we concluded that the review of all exacerbations during the 12-year follow-up period is justifiable because it provides a larger review of prior adverse outcomes. Exacerbation history was excluded from the regression analyses because exacerbations before baseline visit or after follow-up visit were not available in this study. As a result, the association between prior exacerbations and future exacerbations could not be examined.

Our cohort included patients referred to respiratory specialists for diagnosis of asthma. Therefore, it is possible that the proportion of patients with mild asthma is lower and patients with severe asthma is high in our cohort, possibly affecting the results. However, using the American Thoracic Society/European Respiratory Society Task Force definition,^37^ only 5.9% of the study population had severe asthma,^38^ which is in line with population-based studies.^39^^,^^40^

The GINA Report states that having any of the listed 21 asthma exacerbation risk factors (Table E2) increases the patient’s risk of exacerbations, even if they have few asthma symptoms. Our findings suggest that although all risk factors should be assessed, when addressing the risk of future exacerbations in adult-onset asthma patients, single risk factors might lose significance and the cumulative sum of risk factors, and the risk factors most strongly associated with exacerbations should be mainly considered. The prognostic factors should be identified, but the prevention of future exacerbations should be centered around factors that can be affected by clinicians or by the patients themselves (ie, predictive factors). Because our results showed GERD to be the only statistically significant risk factor after adjustments, the listed risk factors could potentially be seen as common characteristics of adult-onset asthma patients rather than actual risk factors for exacerbations. Clinical practice should concentrate on assessing the patient’s risk factor load and preventing future exacerbations with optimal pharmacotherapy of asthma and comorbidities, as well as providing proper patient guidance and encouragement to make healthy lifestyle choices.

Because all patients were prescribed ICS, targeted treatment that was based on type 2 biomarkers was not provided, so we were unable to study asthma biomarkers from a predict-and-prevent standpoint with regard to ideal asthma medication in the prevention of future exacerbations. New studies on asthma biomarkers in adult-onset asthma are needed.

Taken together, asthma exacerbation risk factors are very common in patients with adult-onset asthma. Most patients have several risk factors, and the value of identifying a single risk factor may be low. Patients with multiple risk factors and/or GERD require special attention in clinical practice.

**Key messages**

• Adult-onset asthma patients have multiple risk factors for exacerbations, whereas the value of a single risk factor may be low.
• Patients with multiple risk factors and/or GERD require special attention.

## Disclosure statement

Supported by 10.13039/501100006706Tampere Tuberculosis Foundation (Tampere, Finland), the 10.13039/501100008469Finnish Anti-Tuberculosis Association Foundation (Helsinki, Finland), the Allergy Research Foundation (Helsinki, Finland), the Väinö and Laina Kivi Foundation (Helsinki, Finland), and The Competitive State Research Financing of the Expert Responsibility Area of 10.13039/501100010600Tampere University Hospital (Tampere, Finland). H.K. is funded by the Hermann Krefting Foundation; his work is also supported by Swedish Heart–Lung Foundation, the Swedish Asthma & 10.13039/501100013499Allergy Foundation, ALF agreement ALFGBG-966075 (a grant from the Swedish state under an agreement between the Swedish government and county councils), and the 10.13039/501100004359Swedish Research Council (2022-01022).

Disclosure of potential conflict of interest: H. Kankaanranta reports personal fees for lectures and consulting from AstraZeneca, Boehringer-Ingelheim, Chiesi Pharma, GSK, MSD, Novartis, Orion Pharma, and Sanofi Genzyme, outside the submitted work. L. Lehtimäki reports personal fees for lectures and consulting from ALK, AstraZeneca, Berlin Chemie, Boehringer-Ingelheim, Chiesi, GSK, Novartis, Orion Pharma, and Sanofi Genzyme outside the submitted work. P. Ilmarinen is employed by GSK Finland as a medical advisor. The rest of the authors declare that they have no relevant conflicts of interest.

## Supplementary data

Supplementary Fig E1

Supplementary Fig E2
