# Supplementary material for: Risk factors for asthma exacerbations
Source: J Allergy Clin Immunol Glob. 2025 Jun 21;4(3):100520. doi: 10.1016/j.jacig.2025.100520 (PMC12281877; doi:10.1016/j.jacig.2025.100520)
Supplement: Supplementary Fig E1 [file mmc1.docx]

**Figure E1. Flow-chart of the study^(E1)^**

**Data collection from:** Primary care, occupational and private healthcare, hospital inpatient & outpatient, social insurance institution

**+**

**Asthma related events & medication:** Follow-up visits, exacerbations & unscheduled visits, hospitalizations, medication use

Patients lost to follow-up n=53

Dead n=22 Could not be reached n=9 Refused; comorbidities n=5 Refused; other reasons n=17

Refused: comorbidities n=5

Refused: other reasons n=17

Study population included in the analysis:

Patients at follow-up n=203

Seinäjoki Adult Asthma Study total cohort n=260

**1999 - 2002**

**2012 - 2013**

Patients excluded:

Childhood asthma n=2 Consent withdrawn n=2

Consent withdrawal n=2

**Diagnosis of asthma**

**Follow-up visit**

Asthma control, AQ20, Asthma test, Spirometry, FeNO, PEF-follow-up, blood eosinophils, IgE

Background information:

Asthma-specific questions, medication, life-style factors, socioeconomic factors

**Diagnostic studies**

Spirometry, PEF-follow-up, other respiratory physiology measurements, laboratory, Prick-tests, AQ20, background data, grounds for diagnosis

PEF=peak expiratory flow, AQ20=airways questionnaire 20, FeNO=fractional exhaled nitric oxide, IgE=immunoglobulin E
