# Supplementary material for: Risk factors for asthma exacerbations
Source: J Allergy Clin Immunol Glob. 2025 Jun 21;4(3):100520. doi: 10.1016/j.jacig.2025.100520 (PMC12281877; doi:10.1016/j.jacig.2025.100520)
Supplement: Supplementary Fig E2 [file mmc2.docx]

**Figure E2. Individual asthma exacerbation risk factor scores**

**
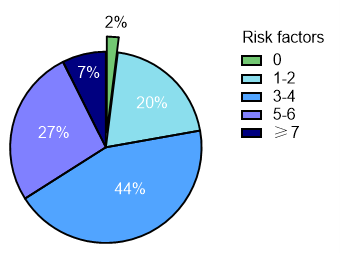
**

Figure E2. The distribution of individual asthma exacerbation risk factor scores.
