# Supplementary material for: Risk factors for asthma exacerbations
Source: J Allergy Clin Immunol Glob. 2025 Jun 21;4(3):100520. doi: 10.1016/j.jacig.2025.100520 (PMC12281877; doi:10.1016/j.jacig.2025.100520)
Supplement: Supplementary Tables [file mmc3.docx]

**Risk factors for asthma exacerbations**

Lauri Nordman^a^, MD, Iida Vähätalo^a,b^, PhD, Leena E. Tuomisto^a,b^, MD, PhD, Onni Niemelä^a,c^, MD, PhD, Minna Tommola^d^, MD, PhD, Lauri Lehtimäki^a,e^, MD, PhD, Pinja Ilmarinen^a,b^, PhD, Hannu Kankaanranta^a,b,f^, MD, PhD

^a^Faculty of Medicine and Health Technology, Tampere University, Tampere, Finland

^b^Department of Respiratory Medicine, Seinäjoki Central Hospital, Seinäjoki, Finland

^c^Department of Laboratory Medicine, Seinäjoki Central Hospital, Seinäjoki, Finland

^d^Department of Respiratory Medicine, Hospital Nova of Central Finland, Jyväskylä, Finland

^e^Allergy Centre, Tampere University Hospital, Tampere, Finland

^f^Krefting Research Centre, Institute of Medicine, Department of Internal Medicine and Clinical Nutrition, University of Gothenburg, Gothenburg, Sweden

**Supplementary material: E-tables**

**Table E1. Inclusion and exclusion criteria (SAAS)^(E1)^**

| Inclusion criteria | - A diagnosis of new-onset asthma made by a respiratory specialist  - Diagnosis confirmed by at least one of the following objective lung function measurements:   - FEV_1_ reversibility in spirometry of at least 15% and 200 mL after 400 µg of salbutamol - Diurnal variability (⩾20% on at least three days) or repeated reversibility (⩾15%/60 l/min on at least three occasions) during a two-week PEF monitoring - A significant decrease in FEV_1_ (15%) or PEF (20%) in to exercise or allergen challenge test - A significant reversibility in FEV_1_ (at least 15% and 200 mL) or mean PEF (at least 20%) in response to a trial with oral or inhaled glucocorticoids   ^-^ Symptoms of asthma  ^-^ Age ≥15 years |
| --- | --- |
| Exclusion criteria | - Physical or mental inability to provide signed informed consent  - Diagnosis of asthma below the age of 15 years  - Of note:   - Patients with comorbidities, either other lung disease or any other significant disease, were not excluded - Patients were not excluded because of smoking, alcohol use or any other lifestyle factor - Respiratory symptoms or any other disease during childhood was not a reason to exclude patients, but a diagnosis of asthma at age <15 years was an exclusion criteria |

FEV_1_= forced expiratory volume in one second, PEF= peak expiratory flow, SAAS= Seinäjoki Adult Asthma Study.

**Table E2. Asthma exacerbation risk factors addressed by GINA Main Report 2023^(E2)^**

| Factors that increase the patient`s risk of exacerbations even if they have few asthma symptoms | |
| --- | --- |
| Medications | High SABA use (≥3 x 200-dose canisters/year) |
|  | Inadequate ICS |
|  | Not prescribed ICS |
|  | Poor adherence |
|  | Incorrect inhaler technique |
| Other medical conditions | Obesity |
|  | Chronic rhinosinusitis |
|  | GERD |
|  | Confirmed food allergy |
|  | Pregnancy |
| Exposures | Smoking |
|  | E-cigarettes |
|  | Allergen exposure if sensitized |
|  | Air pollution |
| Psychosocial | Major psychological or socioeconomic problems |
| Lung function | Low FEV_1_ (especially <60% predicted) |
|  | High BD responsiveness |
| Type 2 inflammatory markers | Higher blood eosinophils |
|  | Elevated FeNO (in adults with allergic asthma taking ICS) |
| Exacerbation history | Ever intubated or in intensive care unit for asthma |
|  | ≥1 severe exacerbation in the last 12 months |

GINA=Global Initiative for Asthma, SABA= short-acting β_2_-agonist, ICS=inhaled corticosteroid, GERD=gastroesophageal reflux disease, e-cigarettes=electronic cigarettes, FEV_1_=forced expiratory volume in 1 second, BD=bronchodilator, FeNO=fractional exhaled nitric oxide

**Table E3. Asthma exacerbation risk factor definitions**

| **Risk factors listed in GINA 2023 Main Report^(E2)^** | **Definitions and/or results of GINA reference articles** | **GINA reference** | **Definition used in this study** |
| --- | --- | --- | --- |
|  |  |  |  |
| **Medication** |  |  |  |
| *High SABA use (≥3 x 200-dose canisters/year associated with increased risk of exacerbations; increased mortality particularly if ≥1 canister per month)* | Collection of more than two SABA canisters in a 1-year baseline period | Nwaru BI, et al. 2020 (E3) | Average use of ≥3 150-dose canisters/year during the follow-up period *^a^* |
|  | Higher mean daily salbutamol use (per two actuations/day), higher days of salbutamol use (per 2 days in 2 weeks) and higher maximal 24-h use (per two actuations/day) were associated with future severe exacerbations. | Patel M, et al. 2013 (E4) |  |
|  | Risk of asthma death begins to escalate drastically at about 1.4 canisters (of 20,000 μg each) per month of inhaled ß-agonist. | Suissa S, et al. 1994 (E5) |  |
|  | Use of 3 or more SABA canisters over 12 months | Stanford RH, et al. 2012 (E6) |  |
| *Inadequate ICS ^b^* | <1 metered dose inhalers of beclomethasone per month over  1-year period | Ernst P, et al. 1992 (E7) | Average daily ICS use of less than 333μg/day during the follow-up period *^a^* |
| *Not prescribed ICS* | Patient has not been prescribed ICS for asthma | Ernst P, et al. 1992 (E7) | ICS was prescribed to all patients included in this study |
| *Poor adherence* | Sufficient use of ICS provides better overall control of asthma and less likelihood of a sudden overwhelming attack. | Ernst P, et al. 1992 (E7) | <80 % adherence to prescribed ICS medication during the follow-up period *^a^* |
| *Incorrect inhaler technique* | Checklist of critical inhalation technique errors: * Failure to remove the cap *Actuation against teeth, lips, or tongue *Activation after end of inhalation *Stopping inhalation immediately after firing * Inhalation through nose whilst and after actuation | Melani AS, et al. 2011 (E8) | Proper inhaler technique was demonstrated and checked at the beginning of the inhaler`s use by a respiratory nurse. Still, proper use was not systematically reviewed. |
|  |  |  |  |
|  |  |  |  |
| **Other medical conditions** |  |  |  |
| *Obesity* | Obesity defined as BMI ≥ 30 | Fitzpatrick S, et al. 2012 (E9) | BMI ≥ 30 at follow-up |
|  | BMI was positively associated with exacerbation frequency. | Denlinger LC, et al. 2017 (E10) |  |
| *Chronic rhinosinusitis* | Chronic rhinosinusitis was associated with exacerbation frequency. | Denlinger LC, et al. 2017 (E10) | Patient meets at least one of the following criteria: *Polyps mentioned at least once in medical records during the follow-up period *Pain or pressure sensation on the face during the follow-up period  *Reduction of sense of smell or taste during the follow-up period *Continuously runny nose at follow-up |
| *GERD* | Gastroesophageal reflux was associated with exacerbation frequency. | Denlinger LC, et al. 2017 (E10) | Treated dyspepsia (H2 blocker or PPI) at follow-up |
| *Confirmed food allergy ^c^* | An sIgE-mediated food allergy requires both the presence of sensitization and the development of specific signs and symptoms on exposure to that food | Burks AW, et al. 2012 (E11) | Sensitization to a foodstuff at follow-up |
| *Pregnancy* | Pregnancy after original asthma diagnosis | Murphy VE, et al. 2006 (E12) | Pregnancy during the follow-up period |
|  |  |  |  |
|  |  |  |  |
| **Exposures** |  |  |  |
| *Smoking* | Risk for exacerbations is higher amongst ever smokers compared to never smokers. | Osborne ML, et al. 2007 (E13) | Ever smoker  (self-reported at baseline and follow-up) |
| *E-cigarettes* | E-cigarette use increases the probability for school absences due to asthma. | Cho JH, et al. 2016 (E14) | No data |
| *Allergen exposure if sensitized* | Skin-prick test positivity with ownership of a cat or dog was identified as a risk factor for exacerbations. | Osborne ML, et al. 2007 (E13) | Sensitization to owned pet  based on allergen specific IgE antibody levels at follow-up (pet ownership self-reported) *^d^* |
| *Air pollution* | Children’s hospital admissions and ED visits for asthma were positively associated with a short-term 10μg/m^3^ increase in PM_2.5_. | Lim H, et al. 2016 (E15) | No data |
|  | Air pollutants were associated with significantly increased risks of asthma ERVs and hospitalizations (O_3_, CO, NO_2_, SO_2_, PM_2.5_ and PM_10_). | Zheng XY, et al. 2015 (E16) |  |
|  | Short-term exposures to high NO_2_ concentrations (>12.37μg/m^3^) were significantly associated with asthma-related ED visits in children and adults. | Mazenq J, et al. 2017 (E17) |  |
|  | Pollutants NO_2_, O_3_, SO_2_ and PM_2.5_ were associated with increases in daily rescue medication use. | Su JG, et al. 2022  (E18) |  |
|  |  |  |  |
|  |  |  |  |
| **Psychosocial** |  |  |  |
| *Major psychological or socioeconomic problems* | Psychosis, alcohol/drug abuse, financial/employment problems and learning difficulties were associated with increased risk of asthma death. | Sturdy PM, et al 2002 (E19) | Patient meets at least one of the following criteria: * Heavy alcohol use, assessed at follow-up *^e^* * Diagnosed schizophrenia * Working age but unemployed, assessed at follow-up  * Didn`t use medication due to financial problems, assessed at follow-up |
|  | Evidence suggestive of increased exacerbation rates among the lowest socioeconomic status group compared to the highest was found. SES included income, poverty, education, employment status and healthcare insurance coverage. | Redmond C, et al. 2022 (E20) |  |
|  |  |  |  |
|  |  |  |  |
| **Lung function** |  |  |  |
| *Low FEV₁* | Risk for acute episodes increases as the FEV₁% predicted decreases (compared groups <60%, 60-80% and >80%). | Osborne ML, et al. 2007 (E13) | FEV₁ < 60 % predicted at the time of the asthma diagnosis (pre- or post-bronchodilator) |
|  | A progressive decrease in the proportion of individuals reporting an attack was associated with increasing decile of FEV_1_%. (FEV_1_ values of >80%, 60-80%, <60%; and >100%, 80-100%, <80%). | Fuhlbrigge AL, et al. 2001 (E21) |  |
| *High BD responsiveness* | BD responsiveness was positively associated with exacerbation frequency for every 10% increase. | Denlinger LC, et al. 2017 (E10) | An increase of ≥ 200ml or ≥ 12% in FEV_1_ or FVC between pre- and post-bronchodilator values during the follow-up period |
|  | In adulthood, eosinophil count was found to be significantly correlated to histamine responsiveness and symptom score while an inverse correlation was found between eosinophil count and FEV_1_% predicted. | Ulrik CS, et al. 1995 (E22) |  |
|  | FEV_1_ bronchodilator responsiveness was the most important characteristic distinguishing difficult- from easy-to-control asthma. | Pongracic JA, et al. 2016 (E23) |  |
|  |  |  |  |
|  |  |  |  |
| **Type 2 inflammatory markers** |  |  |  |
| *Higher blood eosinophils* | Blood eosinophils were positively associated with exacerbation frequency. | Denlinger LC, et al. 2017 (E10) | Blood eosinophils  ≥0.3x10⁹/l at follow-up |
|  | An increased risk of mild exacerbation was associated with blood eosinophil count >0.4x10⁹/l. | Belda J, et al. 2001 (E24) |  |
|  | In subjects with eosinophilia (>0.45x10⁹/l), the risk of dying from asthma was 7.4 (CI 2.8 to 19.7) greater than in those without eosinophilia. | Ulrik CS, et al. 1995 (E25) |  |
| *Elevated FeNO (in adults with allergic asthma taking ICS)* | FeNO levels >300% of predicted are associated with worsening asthma with ICS withdrawal. Highest versus lowest quartile FeNO levels in asthmatic patients on ICSs were associated with exacerbations requiring OCS courses in a prior year, independent of an asthma control tool and spirometry. | Zeiger RS, et al. 2011 (E26) | FeNO >50ppb at follow-up |
|  |  |  |  |
|  |  |  |  |
| **Exacerbation history** |  |  |  |
| *Ever intubated or in intensive care unit for asthma* | Near fatal asthma cases were more likely than controls to have had prior ICU admissions and prior mechanical ventilation. | Turner MO, et al. 1998 (E27) | Ever intubated or in intensive care unit for asthma based on the medical records (assessed at follow-up) |
| *≥1 severe exacerbation in last 12 months* | Asthma-related ED visit or night of hospitalization in the 3 months prior to study visit | Miller MK, et al. 2007 (E28) | ≥1 unscheduled hospital admissions for asthma or use of OCS for asthma during the follow-up period *^f^* |
|  | Asthma symptoms and/or objective evidence of obstruction outside the normal variation for the patient necessitating (A) a short course (at least 3 days) of OCS, and/or (B) a hospitalization or ED visit requiring systemic corticosteroids | Buelo A, et al. 2018 (E29) |  |

GINA=Global Initiative for Asthma, SABA=short-acting β_2_-agonist, ICS=inhaled corticosteroid, BMI=body mass index, GERD=gastroesophageal reflux disease, H2=histamine receptor 2, PPI=proton-pump inhibitor, sIgE= serum immunoglobulin E, e-cigarettes=electronic cigarettes, ED=emergency department, PM_2.5_=particulate matter (diameter of 2.5 micrometers or smaller), ERV=emergency room visit, O_3_=ozone, CO=carbon monoxide, NO_2_=nitrogen dioxide, SO_2_=sulfur dioxide, PM_10_= particulate matter (diameter of 10 micrometers or smaller), SES=socioeconomic status, FEV_1_=forced expiratory volume in 1 second, BD=bronchodilator, FVC=forced vital capacity, FeNO=fractional exhaled nitric oxide, OCS=oral corticosteroid, ICU=intensive care unit

*^a^* The dispensed doses were obtained from the Finnish Social Insurance Institution that records all purchased medication from any Finnish pharmacy.

*^b^* The criteria for inadequate ICS use is based on the following: Ernst P, et al. (E7) states that the risk for adverse effects related to asthma is lower when the patient has dispensed, on average, ≥1 metered-dose of ICS per month. One metered-dose inhaler contains 200 units of 50μg doses. This constitutes to 333,3μg a day for 30 days.

*^c^* Confirmed food allergy was defined as a sensitization to a food stuff as data concerning food allergy diagnosis and allergy symptoms were unavailable.

*^d^* IgE antibody levels assessed included sensitization to dogs, cats, horses and mice.

*^e^* Heavy alcohol use definition: Heavy alcohol consumption was evaluated by self-reports (according to the US definitions for alcohol consumption by portions/week), laboratory analyses [(gammaglutamyltransferase (GT) and gammaglutamyltransferase-carbohydrate-deficient transferrin-index (GT-CDT)] or by both.

*^f^* OCS use was self-reported and hospital admissions were gathered from the medical records.

**Table E4. Exacerbation frequency and OCS use by categorical risk factor scores**

| Asthma exacerbation risk factor score | | | | | | | | |
| --- | --- | --- | --- | --- | --- | --- | --- | --- |
|  | 0 | 1 | 2 | 3 | 4 | 5 | 6 | 7 |
| Patients | 4  (2.0) | 17  (8.4) | 51  (25.1) | 62  (30.5) | 45  (22.2) | 21  (10.3) | 3  (1.5) | - |
| Patients with exacerbations (>1) during the follow-up | 0  (-) | 6  (35.3) | 10  (19.6) | 22  (35.5) | 15  (33.3) | 13  (61.9) | 2  (66.7) | - |
| Exacerbations during the follow-up | 0  (-) | 0  (0-2.0) | 0  (-) | 0  (0-1.0) | 0  (0-1.0) | 1  (0-3.5) | 3  (0-(-)) | - |
| OCS mg/year | 0  (0-30) | 0  (0-85) | 8  (0-93) | 72  (0-181) | 133  (12-324) | 142  (23-272) | 249  (167-(-)) | - |
| OCS mg/12 years | 0  (0-375) | 0  (0-1065) | 110  (0-1200) | 860  (0-2258) | 1700  (150-3860) | 1800  (280-3150) | 3000  (1800-(-)) | - |

OCS=oral corticosteroid

Data was presented as n (%) and median (interquartile range).

Asthma exacerbation defined as: An unplanned healthcare visit with asthma exacerbation mentioned in the medical records by the clinician.

The dispensed OCS doses were obtained from the Finnish Social Insurance Institution that records all purchased medication from any Finnish pharmacy.

**Table E5. Binominal univariate regression analyses for categorical risk factors**

|  | **Exacerbations ≥1** |  | **Exploratory analysis:**  **Unplanned respiratory- related healthcare visits ≥1** |  |
| --- | --- | --- | --- | --- |
|  | **OR (95% CI)** | **P-value** | **OR (95% CI)** | **P-value** |
| **Medications** | 0.773 (0.430-1.391) | 0.391 | 1.232 (0.640-2.373) | 0.533 |
| **Other medical conditions** | 2.121 (0.949-4.738) | 0.067 | 1.674 (0.786-3.565) | 0.181 |
| **Exposures** | 1.329 (0.735-2.401) | 0.347 | 1.591 (0.826-3.064) | 0.165 |
| **Psychosocial** | 0.914 (0.462-1.808) | 0.796 | 1.505 (0.670-3.382) | 0.322 |
| **Lung function** | 1.199 (0.620-2.317) | 0.590 | 0.759 (0.368-1.566) | 0.456 |
| **Type 2 inflammatory markers** | 1.087 (0.525-2.249) | 0.822 | 1.904 (0.746-4.862) | 0.178 |

Asthma exacerbation defined as: An unplanned healthcare visit with asthma exacerbation mentioned in the medical records by the clinician.

Unplanned respiratory-related healthcare visit defined as: Unplanned healthcare visits regarding upper respiratory tract infections (URTI) and/or asthma exacerbations based on medical records.

All univariate regression analyses regarding asthma exacerbation risk factors were conducted with patients without the risk factor in question as the reference group.

**Table E6. Binominal multivariable logistic regression analyses: Exacerbations**

|  | **OR (95% CI)** | **P-value** |
| --- | --- | --- |
| **Female** | 1.056 (0.555-2.010) | 0.868 |
| **>50 years old** | 2.081 (0.993-4.363) | 0.052 |
| **GERD** *^a^* | 4.846 (1.561-15.049) | **0.006** |
| **Obesity *^b^*** | 1.029 (0.545-1.946) | 0.929 |
| **Smoking *^c^*** | 1.282 (0.668-2.462) | 0.455 |

GERD=gastroesophageal reflux disease

*^a^* Patients without GERD served as the reference group.

*^b^* Obesity defined as BMI≥30

*^c^* Smoking defined as ever smoker

**Table E7. Unplanned respiratory-related healthcare visits by categorical risk factor scores**

| Asthma exacerbation risk factor score | | | | | | | | |
| --- | --- | --- | --- | --- | --- | --- | --- | --- |
|  | 0 | 1 | 2 | 3 | 4 | 5 | 6 | 7 |
| Patients | 4  (2.0) | 17  (8.4) | 51  (25.1) | 62  (30.5) | 45  (22.2) | 21  (10.3) | 3  (1.5) | - |
| Patients with URHVs (>1) during the follow-up | 1  (25.0) | 11  (64.7) | 34  (66.7) | 50  (80.6) | 38  (84.4) | 19  (90.5) | 3  (100) | - |
| URHVs during the follow-up | 0.0  (0.0-0.75) | 2.0  (0.0-7.0) | 2.0  (0.0-9.0) | 4.5  (1.0-9.25) | 3,0  (1,0-9,5) | 4.0  (1.5-12.5) | 18.0  (12.0-) | - |

URHV=Unplanned respiratory-related healthcare visit

Data was presented as n (%) and median (interquartile range).

Unplanned respiratory visits defined as: Unplanned healthcare visits regarding upper respiratory tract infections (URTI) and/or asthma exacerbations based on medical records.

**Table E8. Unplanned respiratory-related healthcare visits by individual risk factor scores**

| Asthma exacerbation risk factor score | | | | | | | | | |
| --- | --- | --- | --- | --- | --- | --- | --- | --- | --- |
|  | 0 | 1 | 2 | 3 | 4 | 5 | 6 | 7 | ≥8 |
| Patients | 4  (2.0) | 14  (6.9) | 27  (13.3) | 47  (23.2) | 42  (20.7) | 33  (16.3) | 21  (10.3) | 11  (5.4) | 4  (2.0) |
| Patients with URHVs (>1) during the follow-up | 1  (25.0) | 8  (57.1) | 19  (70.4) | 38  (80.9) | 30  (71.4) | 30  (90.9) | 15  (71.4) | 11  (100.0) | 4  (100.0) |
| URHVs during the follow-up | 0.0  (0.0-0.75) | 1.5  (0.0-7.0) | 4.0  (0.0-9.0) | 3.0  (1.0-10.0) | 2.5  (0.0-8.0) | 8.0  (1.0-11.0) | 3.0  (0.0-8.0) | 3.0  (1.0-9.0) | 15.0  (3.75-20.25) |

URHV=Unplanned respiratory-related healthcare visit

Data was presented as n (%) and median (interquartile range).

Unplanned respiratory-related healthcare visit defined as: Unplanned healthcare visits regarding upper respiratory tract infections (URTI) and/or asthma exacerbations based on medical records.

**Table E9. Binominal multivariable logistic regression analyses: Unplanned respiratory- related healthcare visits**

|  | **OR (95% CI)** | **P-value** |
| --- | --- | --- |
| **Female** | 1.808 (0.888-3.683) | 0.103 |
| **>50 years old** | 1.240 (0.588-2.617) | 0.572 |
| **Chronic rhinosinusitis *^a^*** | 2.520 (1.269-5.008) | **0.008** |
| **Obesity *^b^*** | 1.102 (0.541-2.246) | 0. 789 |
| **Smoking *^c^*** | 1.775 (0.865-3.641) | 0.118 |

*^a^* Patients without chronic rhinosinusitis served as the reference group.

*^b^* Obesity defined as BMI≥30

*^c^* Smoking defined as ever smoker

**E-supplement references**

1. Kankaanranta H, Ilmarinen P, Kankaanranta T, Tuomisto LE. Seinäjoki adult asthma study (SAAS): A protocol for a 12-year real-life follow-up study of new-onset asthma diagnosed at adult age and treated in primary and specialized care. NPJ Prim Care Respir Med 2015; 25: 15042.
2. Global Initiative for Asthma (GINA). Global Strategy for Asthma Management and Prevention, 2023. Available from: <http://ginasthma.org/>
3. Nwaru BI, Ekstrom M, Hasvold P, Wiklund F, Telg G, Janson C. Overuse of short-acting beta2-agonists in asthma is associated with increased risk of exacerbation and mortality: a nationwide cohort study of the global SABINA programme. Eur Respir J 2020; 55: 1901872.
4. Patel M, Pilcher J, Reddel HK, Pritchard A, Corin A, Helm C, et al. Metrics of salbutamol use as predictors of future adverse outcomes in asthma. Clinical & Experimental Allergy 2013; 43: 1144–1151.
5. Suissa S, Ernst P, Boivin JF, Horwitz RI, Habbick B, Cockroft D, et al. A cohort analysis of excess mortality in asthma and the use of inhaled beta-agonists. AM J Respir Crit Cre Med 1994; 149: 604–610.
6. Stanford RH, Shah MB, D´Souza AO, Dhamane AD, Schatz M. Short-acting β-agonist use and its ability to predict future asthma-related outcomes. Annals of allergy, Asthma & Immunology 2012; 109: 403–407.
7. Ernst P, Spitzer WO, Suissa S, Cockcroft D, Habbick B, Horwitz RI, et al. Risk of fatal and near-fatal asthma in relation to inhaled corticosteroid use. JAMA 1992; 268: 3462–3464.
8. Melani AS, Bonavia M, Cilenti V, Cinti C, Lodi M, Martucci P, et al. Inhaler mishandling remains common in real life and is associated with reduced disease control. Respir Med 2011; 105: 930–938.
9. Fitzpatrick S, Joks R, Silverberg JI. Obesity is associated with increased asthma severity and exacerbations, and increased serum immunoglobulin E in inner-city adults. Clinical & Experimental Allergy 2012; 42: 747–759.
10. Denlinger LC, Phillips BR, Ramratnam S, Ross K, Bhakta NR, Cardet JC, et al. Inflammatory and Comorbid Features of Patients with Severe Asthma and Frequent Exacerbations. Am J Respir Crit Care Med 2017; 195: 302–313.
11. Burks AW, Tang M, Sicherer S, Muraro A, Eigenmann PA, Ebisawa M, et al. ICON: food allergy. Journal of Allergy & Clinical Immunology 2012; 129: 906–920.
12. Murphy VE, Clifton VL, Gibson PG. Asthma exacerbations during pregnancy: incidence and association with adverse pregnancy outcomes. Thorax 2006; 61: 169–176.
13. Osborne ML, Pedula KL, O’Hollaren M, Ettinger KM, Stibolt T, Buist AS, et al. Assessing future need for acute care in adult asthmatics: the Profile of Asthma Risk Study: a prospective health maintenance organization-based study. Chest 2007; 132: 1151–1161.
14. Cho JH, Paik SY. Association between electronic cigarette use and asthma among high school students in South Korea. PloS One 2016; 11: e0151022.
15. Lim H, Kwon HJ, Lim JA, Choi JH, Ha M, Hwang SS, et al. Short-term effect of fine particulate matter on children’s hospital admissions and emergency department visits for asthma: A systematic review and meta-analysis. J Prev Med Public Health 2016; 49: 205–219.
16. Zheng XY, Ding H, Jiang LN, Chen SW, Zheng JP, Qiu M, et al. Association between air pollutants and asthma emergency room visits and hospital admissions in time series studies: A systematic review and meta-analysis. PloS One 2015; 10: e0138146.
17. Mazenq J, Dubus JC, Gaudart J, Charpin D, Viudes G, Noel G. City housing atmospheric pollutant impact on emergency visit for asthma: A classification and regression tree approach. Respir Med 2017; 132: 1–8.
18. Su JG, Barrett MA, Combs V, Henderson K, Van Sickle D, Hogg C, et al. Identifying impacts of air pollution on subacute asthma symptoms using digital medication sensors. Int J Epidemiol. 2022 Feb 18;51(1):213-224.
19. Sturdy PM, Victor CR, Anderson HR, Bland JM, Butland BK, Harrison BD, et al. Psychological, social and health behaviour risk factors for deaths certified as asthma: a national case-control study. Thorax 2002; 57: 1034–1039.
20. Redmond C, Akinoso-Imran AQ, Heaney LG, Sheikh A, Kee F, Busby J. Socioeconomic disparities in asthma health care utilization, exacerbations, and mortality: A systematic review and meta-analysis. J Allergy Clin Immunol. 2022 May;149(5):1617-1627.
21. Fuhlbrigge AL, Kitch BT, Paltiel AD, Kuntz KM, Neumann PJ, Dockery DW, et al. FEV1 is associated with risk of asthma attacks in a pediatric population. J Allergy Clin Immunol 2001; 107: 61–67.
22. Ulrik CS. Peripheral eosinophil counts as a marker of disease activity in intrinsic and extrinsic asthma. Clin Exp Allergy 1995; 25: 820–827.
23. Pongracic JA, Krouse RZ, Babineau DC, Zoratti EM, Cohen RT, Wood RA, et al. Distinguishing characteristics of difficult-to-control asthma in inner-city children and adolescents. J Allergy Clin Immunol 2016; 138: 1030–1041.
24. Belda J, Giner J, Casan P, Sanchis J. Mild exacerbations and eosinophilic inflammation in patients with stable, well-controlled asthma after 1 year of follow-up. Chest 2001; 119: 1011–1017.
25. Ulrik CS, Frederiksen J. Mortality and markers of risk of asthma death among 1,075 outpatients with asthma. Chest 1995; 108: 10–15.
26. Zeiger RS, Schatz M, Zhang F, Crawford WW, Kaplan MS, Roth RM, et al. Elevated exhaled nitric oxide is a clinical indicator of future uncontrolled asthma in asthmatic patients on inhaled corticosteroids. Journal of Allergy and Clinical Immunology 2011; 128: 412-414.
27. Turner MO, Noertjojo K, Vedal S, Bai T, Crump S, Fitzgerald JM. Risk factors for near-fatal asthma. A case-control study in hospitalized patients with asthma. Am J Respir Crit Care Med 1998; 157: 1804–1809.
28. Miller MK, Lee JH, Miller DP, Wenzel SE; TENOR Study Group. Recent asthma exacerbations: a key predictor of future exacerbations. Respir Med 2007; 101: 481–489.
29. Buelo A, McLean S, Julious S, Flores-Kim J, Bush A, Henderson J, et al. At-risk children with asthma (ARC): a systematic review. Thorax 2018; 73: 813–824.
